# Comparison of hepatic arterial infusion chemotherapy with mFOLFOX vs. first-line systemic chemotherapy in patients with unresectable intrahepatic cholangiocarcinoma

**DOI:** 10.3389/fphar.2023.1234342

**Published:** 2023-09-05

**Authors:** Zhenyun Yang, Yizhen Fu, Weijie Wu, Zili Hu, Yangxun Pan, Juncheng Wang, Jinbin Chen, Dandan Hu, Zhongguo Zhou, Minshan Chen, Yaojun Zhang

**Affiliations:** ^1^ State Key Laboratory of Oncology in South China, Collaborative Innovation Center for Cancer Medicine, Sun Yat-Sen University Cancer Center, Guangzhou, Guangdong, China; ^2^ Department of Liver Surgery, Sun Yat-Sen University Cancer Center, Guangzhou, Guangdong, China

**Keywords:** intrahepatic cholangiocarcinoma, hepatic arterial infusion chemotherapy, systemic chemotherapy, overall survival, progression-free survival, adverse events

## Abstract

**Background:** Systemic chemotherapy (SC) remains the only first-line treatment for unresectable intrahepatic cholangiocarcinoma (iCCA). Hepatic arterial infusion chemotherapy (HAIC) has been recently proven to be effective in managing hepatocellular carcinoma (HCC). Hence, our study aims to investigate the safety and efficacy of HAIC in treating unresectable iCCA patients.

**Methods:** We reviewed 146 patients with unresectable iCCA who had received HAIC or SC between March 2016 and March 2022 in a retrospective manner. Outcomes of patients and safety were compared between the HAIC and SC groups.

**Results:** There were 75 and 71 patients in the HAIC and SC groups, respectively. The median OS in the HAIC and SC groups was 18.0 and 17.8 months (*p* = 0.84), respectively. The median PFS in the HAIC and SC groups was 10.8 and 11.4 months (*p* = 0.59), respectively. However, the HAIC group had significantly longer intrahepatic progression-free survival (IPFS) than the SC group (*p* = 0.035). The median IPFS in the HAIC and SC groups was 13.7 and 11.4 months, respectively. According to the OS (*p* = 0.047) and PFS (*p* = 0.009), single-tumor patients in the HAIC group appeared to benefit more. In addition, the overall incidence of adverse events (AEs) was lower in the HAIC group than that in the SC group.

**Conclusion:** Our study revealed that HAIC was a safe and effective therapeutic regimen for unresectable iCCA with better intrahepatic tumor control when compared to SC. Meanwhile, patients with single tumor were more likely to benefit from HAIC than SC.

## Introduction

Intrahepatic cholangiocarcinoma (iCCA) is the second most frequent primary liver cancer with a poor prognosis and high level of malignancy (Bridgewater et al., 2014; Sirica et al., 2019; Valle et al., 2021). The incidence of iCCA is higher in Thailand and China (6 per 100,000 people) than that in Western Europe and North America (0·35 to 2 per 100,000 people) (Banales et al., 2016; Oh et al., 2022). Over the next 20–30 years, the incidence of iCCA will increase ten-fold worldwide (Rodriguez and Pennington, 2018; Dong et al., 2022). Surgical resection is currently the first-line and curative therapy for iCCA management. However, most iCCA patients are diagnosed at a late stage as a result of the absence of specific clinical symptoms and limited treatment modalities for iCCA (Rizvi and Gores, 2013; Bupathi et al., 2017; Rizvi et al., 2018).

Currently, the first-line systemic chemotherapy (SC) for biliary tract cancer is gemcitabine plus cisplatin (GEMCIS), with a median overall survival (OS) of 11.7 months (Valle et al., 2010). Oxaliplatin plus gemcitabine (GEMOX) is also a common treatment regimen for biliary tract cancer patients in Asia, with a similar median OS compared to GEMCIS (Fiteni et al., 2014; Kim et al., 2019). The FOLFOX regimen may be an option for the palliative treatment of advanced cholangiocarcinoma (Nehls et al., 2002; Caparica et al., 2019; Lamarca et al., 2021).

Hepatic arterial infusion chemotherapy (HAIC) enables the delivery of chemotherapy drugs directly into the liver. Tumors derive most of their nutrients from the arteries, whereas the liver derives nutrients from the portal vein, which may reduce systemic adverse events (AEs) from systemic chemotherapy (Kemeny et al., 1984; Cercek et al., 2020). Meanwhile, previous studies have clarified that HAIC is useful for advanced iCCA and has shown higher tumor control rates compared to systemic chemotherapy (Kasai et al., 2014; Cercek et al., 2020). However, there was no study comparing HAIC with FOLFOX and first-line systemic chemotherapy in relation to patients’ outcomes and AEs.

Herein, the current study compares the clinical outcomes and tumor response of patients with unresectable iCCA treated with HAIC and SC. In addition, the assessment of safety and AEs were also vital in this retrospective study.

## Materials and methods

### Patients’ recruitment and selection criteria

This is a retrospective study, and the study subjects consisted of 146 patients diagnosed with iCCA who were initially treated with HAIC or first-line SC between March 2016 and March 2022 at Sun Yat-sen University Cancer Center, China. Participants were included if they conformed to the following criteria: (Bridgewater et al., 2014) age 18 years old or elder; (Sirica et al., 2019) histopathological evidence confirmation of iCCA; (Valle et al., 2021) confirmed records of primary HAIC or first-line SC; (Oh et al., 2022) an Eastern Cooperative Oncology Group (ECOG) score of 2 or below; and (Banales et al., 2016) complete medical follow-up data. Patients were excluded based on the following exclusion criteria: (Bridgewater et al., 2014) patients with any other malignant tumor and (Sirica et al., 2019) patients who had contraindications to HAIC and SC.

### Treatment procedures

HAIC was performed according to our previously reported protocol (Li et al., 2022). Femoral artery puncture and catheterization were performed on day 1 of the HAIC cycle, and the patient was transferred to the inpatient ward for drug infusion through the hepatic artery. Oxaliplatin was administered at 130 mg/m^2^ from 0 to 2 h on day 1; leucovorin was administered at 400 mg/m^2^ from 2 to 3 h on day 1; fluorouracil was administered at 400 mg/m^2^ from hour 3 on day 1. Infusional fluorouracil was given at 2400 mg/m^2^ over 23 h or 46 h. HAIC cycles were performed every 3 weeks. In the GEMCIS group, each cycle comprised cisplatin (25 mg per square meter of body-surface area), followed by gemcitabine (1,000 mg per square meter), which was administered on days 1 and 8 every 3 weeks. In the GEMOX group, each cycle comprised oxaliplatin (85 mg/m2) on day 1 and gemcitabine (1,000 mg per square meter) between days 1 and 8 every 3 weeks. HAIC or SC was suspended at 24 weeks or because of disease progression, unacceptable toxic effects, or patient’s own choice. As a part of treatment, HAIC or SC may be combined with the PD-1 inhibitor or tyrosine kinase inhibitor according to the needs of the condition and patient’s own choice.

### Data collection

All clinical data were obtained from the medical records of the Sun Yat-sen University Cancer Center. Demographic and clinical characteristics included age, sex, hepatitis infection status, ECOG, aspartate aminotransferase (AST), alanine transaminase (ALT), albumin (ALB), total bilirubin (TBIL), carcinoembryonic antigen (CEA), carbohydrate antigen 19–9 (CA19–9), white blood cell count (WBC), platelet count (PLT), creatinine (CRE), largest tumor size, tumor number, macroscopic vascular invasion, lymph node metastasis, extra-hepatic metastasis, and tumor–node–metastasis (TNM) stages. A summary of demographic and clinical characteristics is presented in Table 1. The blood tests and tumor burdens were measured within 5 days before the treatment. After treatment had been initiated, the radiological response was evaluated by magnetic resonance imaging (MRI) or computed tomography (CT) performed at baseline and every 6 weeks. Response Evaluation Criteria in Solid Tumors (RECIST)1.1 and modified RECIST (mRECIST) were used for evaluating the tumor response (Eisenhauer et al., 2009; Llovet and Lencioni, 2020).

**TABLE 1:** Baseline characteristics of two group patients.

Variable	HAIC group (n = 75)	SC group (n = 71)	*p*-value
Age (years)	54 (28–78)	57 (32–80)	0.152
Gender (men/women)	52/23 (69.3/30.7)	40/31 (56.3/43.7)	0.104
Hepatitis (yes/no)	34/41 (45.3/54.7)	25/46 (35.2/64.8)	0.213
ECOG (1–2/0)	45/30 (60/40)	40/31 (56.3/43.7)	0.654
Preoperative blood tests			
AST (IU/L)	35.8 (14.8–169.1)	30.5 (11.8–174)	0.311
ALT (IU/L)	27.6 (7.4–179.4)	23.7 (8.5–209.2)	0.999
ALB (g/L)	41.5 (25.9–53.5)	41.4 (30.6–48)	0.316
TBIL (umol/L)	12.5 (5.4–69.5)	11.6 (3.8–256)	0.492
CEA (ng/mL)	4.2 (0.3–6,395)	4.6 (0.5–8,952)	0.945
CA19–9(U/mL)	90.1 (1.0–200000)	152 (0.6–200000)	0.531
WBC(10^9^/L)	8.0 (4.4–26.6)	8.4 (4.7–14.8)	0.177
PLT (10^9^/L)	272 (66–490)	232 (81–578)	0.302
CRE(umol/L)	66.4 (30.6–133)	62.5 (30.6–133)	0.683
Tumor burden			
Largest tumor size, cm (>10/≤10)	25/50 (33.3/66.7)	14/57 (19.7/80.3)	0.063
Tumor numbers (single/multiple)	25/50 (33.3/66.7)	22/49 (31/69)	0.762
Macrovascular invasion (yes/no)	23/52 (30.7/69.3)	18/53 (25.4/74.6)	0.475
Lymph node metastasis (yes/no)	51/24 (68/32)	47/24 (66.2/33.8)	0.817
Extrahepatic metastasis (yes/no)	17/58 (22.7/77.3)	24/47 (33.8/66.2)	0.135
TNM stage (III-IV/II)	56/19 (74.7/25.3)	56/15 (78.9/21.1)	0.548
Cycle times	4 (2–8)	3 (2–7)	0.628
Combination therapy (yes/no)	32/43 (42.6/57.3)	26/45 (36.6/63.4)	0.455

Values are presented as the median (range) or n (%).

**Abbreviations**: HAIC, hepatic arterial infusion chemotherapy; SC, systemic chemotherapy; ECOG, Eastern Cooperative Oncology Group; AST, aspartate transaminase; ALT, alanine transaminase; ALB, albumin; TBIL, total bilirubin; CEA, carcinoembryonic antigen; CA19–9, carbohydrate antigen 19–9; WBC, white blood cell; PLT, platelet count; CRE, creatinine; TNM, tumor–node–metastasis.

Overall survival (OS) was defined as the time interval from first-line treatment to cancer-related death. Progression-free survival (PFS) was defined as the interval from first-line treatment to disease progression, iCCA relapse, or the date of death from iCCA or the date of the last follow-up. Intrahepatic progression-free survival (IPFS) was defined as the interval from the first-line treatment to intrahepatic tumor progression, iCCA relapse, or the date of death from iCCA or the date of the last follow-up, regardless of extrahepatic metastasis.

### Statistical analysis

Non-normally distributed data were expressed as medians and ranges. Continuous parametric variables were analyzed by the unpaired Student’s *t*-test, and continuous non-parametric variables were analyzed by the Mann–Whitney *U* test. Categorical data were analyzed by Pearson’s correlation coefficient, chi-squared test with continuity corrections, or Fisher’s exact probability method. Forward LR-based univariate and multivariate Cox regression analyses were conducted to identify independent predictive variables. The OS and PFS were shown by Kaplan–Meier curves, and differences between the groups were compared using the results of the log-rank test. The *p*-value <0.05 was considered statistically significant. All the analyses were performed using SPSS 25.0 software (SPSS Inc., Chicago, IL) and R version 4.0.1.

## Results

### Patient characteristics

**FIGURE 1:**
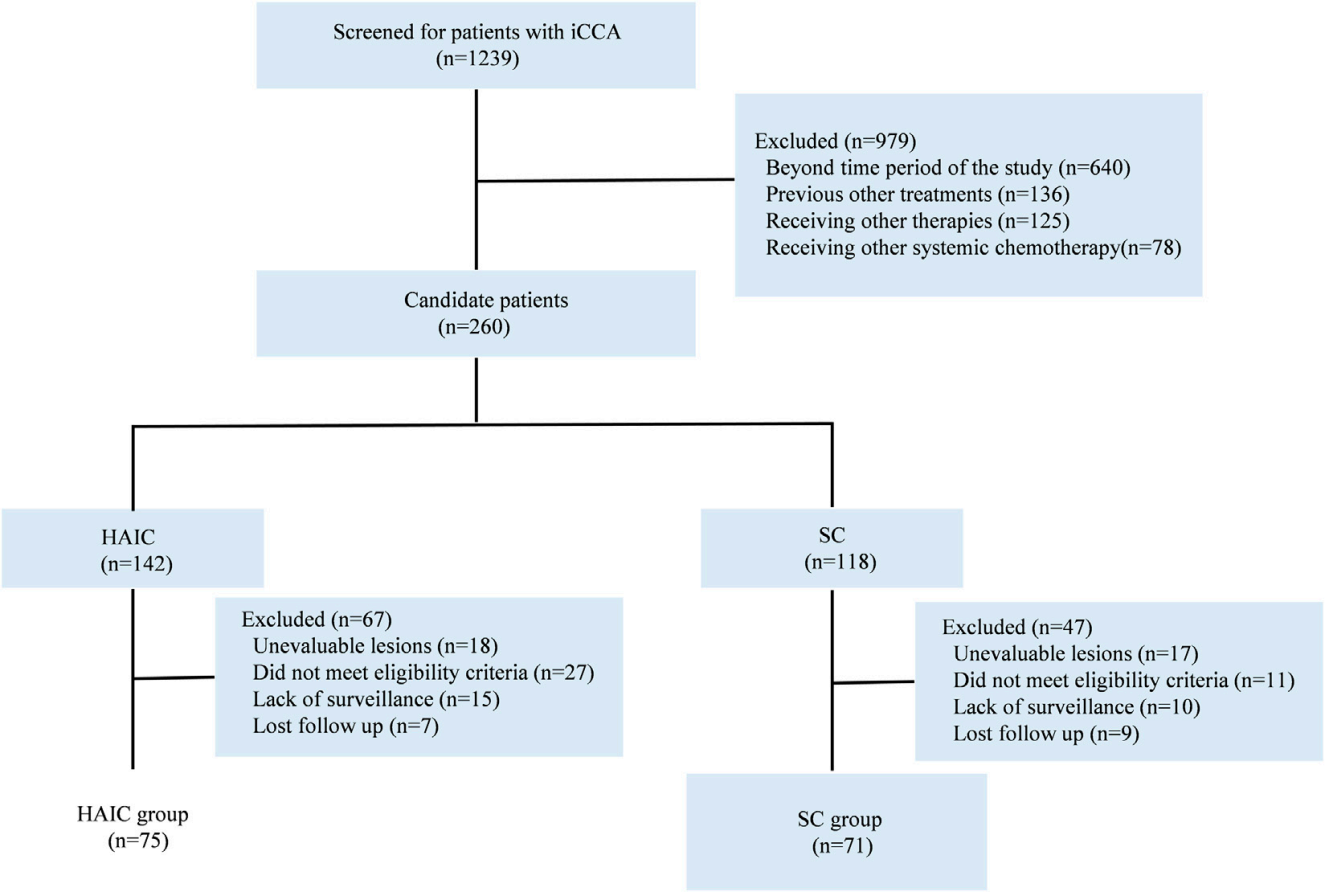
Flowchart for patient inclusion. Abbreviations: iCCA, intrahepatic cholangiocarcinoma; HAIC, hepatic arterial infusion chemotherapy; SC, systemic chemotherapy.

Between March 2016 and March 2022, 146 patients diagnosed with iCCA who initially received HAIC or first-line SC were selected at Sun Yat-sen University Cancer Center, China. There were 75 patients in the HAIC group and 71 patients in the SC group (Figure 1). Detailed characteristics of each group are shown in Table 1. No significant baseline differences existed between the HAIC and SC groups.

In the HAIC group, the median age was 54 years old, 52 patients were male subjects, the largest tumor size of 25 (33.3%) patients was longer than 10 cm, a majority of patients had multiple tumors (66.7%), a total of 23 (30.7%) patients had macrovascular invasion, 51 (68%) patients had lymph node metastasis, and 17 (22.7%) patients had extra-hepatic metastasis. In the SC group, the median age was 57 years old, and 40 patients were male subjects, the largest tumor size of 14 (19.7%) patients was longer than 10 cm, a majority of patients had multiple tumors (69%), a total of 18 (25.4%) patients had macrovascular invasion, 47 (66.2%) patients had lymph node metastasis, and 24 (33.8%) patients had extra-hepatic metastasis. According to characteristics of a tumor, most patients in this study had large tumor burden and advanced iCCA.

### Univariate and multivariate Cox regression analyses in the cohorts

Prognostic factors of all clinical variables were analyzed in univariate analysis. Univariate analyses showed that ECOG, tumor number, extra-hepatic metastasis, and TNM stages were significant risk factors for patients’ OS. Univariate analysis for PFS showed that ECOG, CA19–9, and extra-hepatic metastasis were significant risk factors. More details are described in Table 2. The multivariate Cox proportional analysis revealed that ECOG (*p* < 0.001) and extra-hepatic metastasis (*p* = 0.026) were significant and independent prognostic factors of OS (Table 2). The multivariate Cox proportional analysis revealed that ECOG (*p* < 0.001), CA19–9 (*p* = 0.02), macrovascular invasion (*p* = 0.02), and extra-hepatic metastasis (*p* = 0.001) were significant and independent prognostic factors of PFS (Table 2).

**TABLE 2:** Univariate and multivariate Cox regression analyses of risk factors for overall survival and progression-free survival.

Variable	OS				PFS			
	Univariate		Multivariate		Univariate		Multivariate	
	HR (95% CI)	*p*-value	HR (95% CI)	*p*-value	HR (95% CI)	*p*-value	HR (95% CI)	*p*-value
Age, y (>/≤50)	0.99 (0.59–1.67)	0.96			0.78 (0.51–1.22)	0.28		
Gender (men/women)	1.05 (0.65–1.67)	0.85			1.17 (0.74–1.86)	0.5		
Hepatitis (yes/no)	1.29 (0.82–2.03)	0.27			1.29 (0.82–2.00)	0.27		
ECOG (≥1/0)	13.48 (5.83–31.17)	<0.001	13.18 (5.7–30.5)	<0.001	4.22 (2.39–7.44)	<0.001	4.52 (2.53–8.06)	<0.001
ALB, g/L, (>/≤35)	0.60 (0.29–1.21)	0.16			0.68 (0.35–1.33)	0.26		
TBIL, umol/L, (>/≤17.1)	1.21 (0.70–2.11)	0.49			1.62 (0.94–2.78)	0.08		
CA19–9,U/mL, (>/≤100)	0.98 (0.62–1.54)	0.92			1.68 (1.08–2.59)	0.02	1.69 (1.09–2.62)	0.02
CEA, ng/mL (>5/≤5)	1.54 (0.76–3.10)	0.23			1.21 (0.58–2.51)	0.61		
Largest tumor size (>/≤10 cm)	1.49 (0.93–2.39)	0.09			0.81 (0.51–1.3)	0.39		
Tumor numbers (>1/1)	1.65 (1.05–2.61)	0.03			1.28 (0.81–2.02)	0.29		
Macrovascular invasion (yes/no)	0.77 (0.46–1.29)	0.33			1.55 (0.94–2.56)	0.08	1.79 (1.08–2.99)	0.02
Lymph node metastasis (yes/no)	0.83 (0.51–1.33)	0.43			1.19 (0.75–1.89)	0.47		
Extrahepatic metastasis (yes/no)	1.86 (1.17–2.95)	0.008	1.69 (1.01–2.67)	0.026	2.12 (1.37–3.29)	0.001	2.12 (1.35–3.32)	0.001
TNM stage (III-IV/II)	1.76 (1.0–3.1)	0.05			1.70 (0.97–2.97)	0.06		
Therapy (SC/HAIC)	0.95 (0.61–1.51)	0.84			1.13 (0.72–1.77)	0.59		

*p*-value <0.05 is statistically significant in both univariate and multivariate analyses.

**Abbreviations:** ECOG, Eastern Cooperative Oncology Group; ALB, albumin; TBIL, total bilirubin; CA19–9 carbohydrate antigen 19–9; CEA, carcinoembryonic antigen; TNM, tumor–node–metastasis; SC, systemic chemotherapy; HAIC, hepatic artery infusion chemotherapy.

### Tumor response and patient survival

**FIGURE 2:**
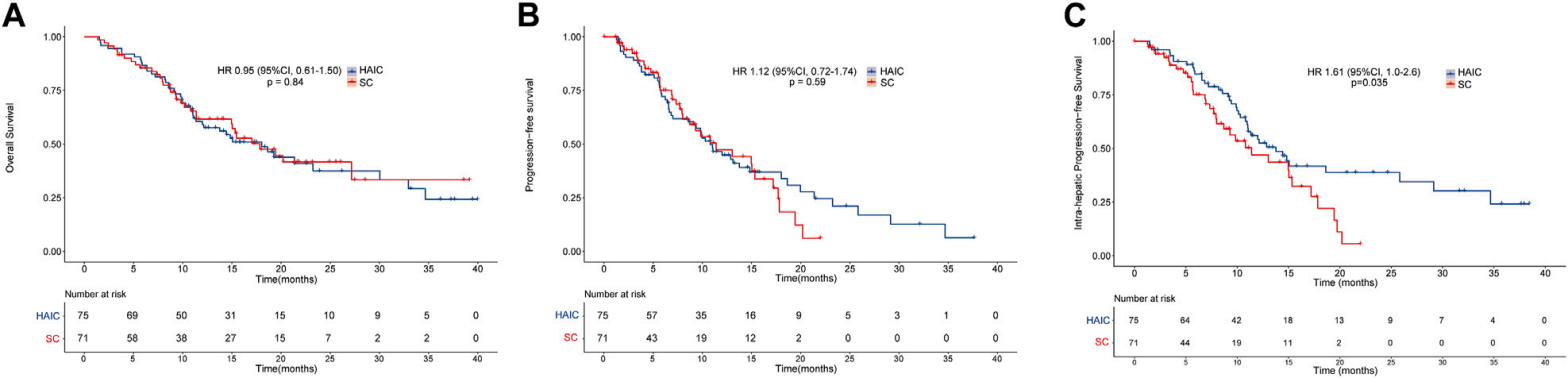
Overall survival and progression-free survival of the two groups of patients. Kaplan–Meier curves of **(A)** overall survival, **(B)** progression-free survival, and **(C)** intrahepatic progression-free survival for patients in the HAIC and SC groups.

The median OS in the HAIC and SC groups was 18.0 and 17.8 months, respectively. Meanwhile, the median PFS times in the HAIC and SC groups were 10.8 and 11.4 months, respectively. There was no significant difference between the two groups in OS (*p* = 0.84; Figure 2A) and PFS (*p* = 0.59; Figure 2B). However, patients in the HAIC group had significantly longer IPFS than patients in the SC group (*p* = 0.035; Figure 2C). The median IPFS in the HAIC and SC groups was 13.7 and 11.4 months, respectively. The median follow-up in the HAIC and SC group was 16.8 and 17.7 months, respectively (Supplementary Figure S1). Patients in the SC group were divided into two subgroups (GEMCIS and GEMOX). GEMCIS and GEMOX were compared with HAIC in OS and PFS (Supplementary Figure S2).

**FIGURE 3:**
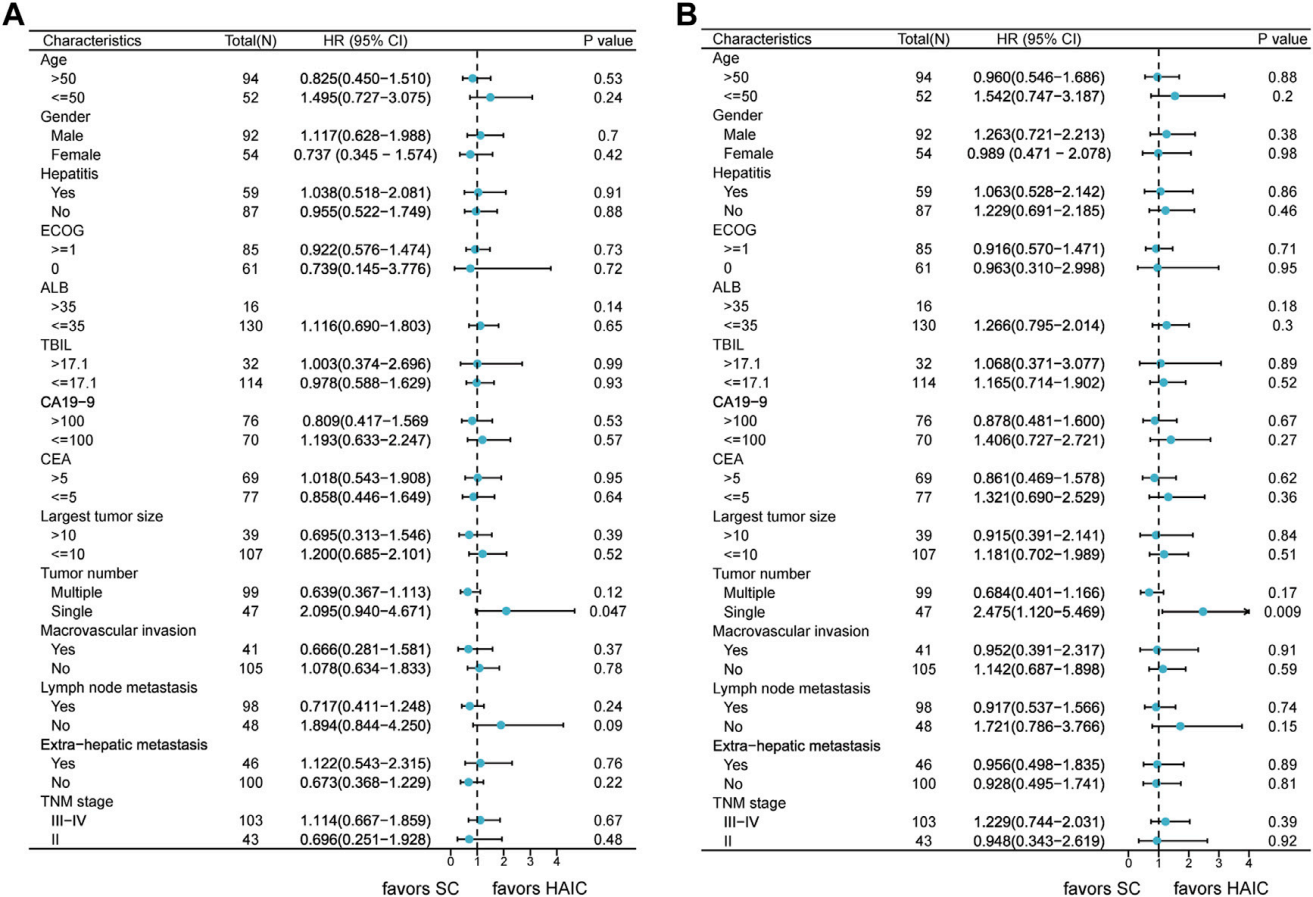
Forest plots of **(A)** overall survival and **(B)** progression-free survival in different patient subgroups. Abbreviations: HR, hazard ratio; CI, confidence interval; ECOG, Eastern Cooperative Oncology Group; ALB, albumin; TBIL, total bilirubin; CA19–9, carbohydrate antigen 19–9; CEA, carcinoembryonic antigen; TNM, tumor–node–metastasis.

**FIGURE 4:**
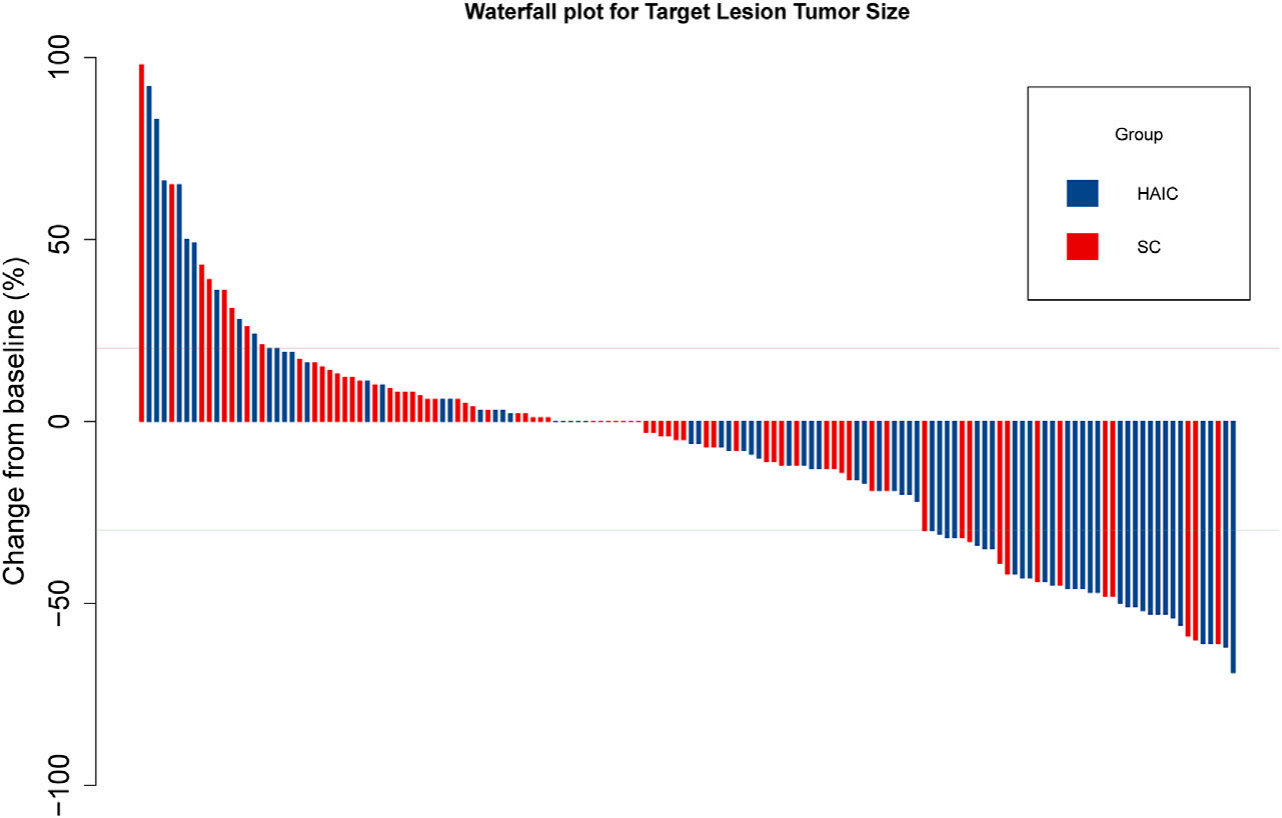
Waterfall plot for tumor size changes in intrahepatic target lesions. Abbreviations: PD, progressive disease; PR, partial response.

The subgroup analyses of OS and PFS are shown in Figure 3. HAIC provided a clinical benefit for OS and PFS in tumor number subgroups. Single-tumor patients appeared to benefit more from it in terms of OS (*p* = 0.047; Supplementary Figure S3A) and PFS (*p* = 0.009; Supplementary Figure S3B). The intrahepatic tumor responses of the patients are shown in Table 3. On the basis of RECIST1.1 and mRECIST criteria, HAIC showed an ORR two times higher than SC (40% vs. 16.9%, *p* = 0.002, RECIST1.1; 45.3% vs. 21.2%, *p* = 0.002, mRECIST). The optimal response for intrahepatic target lesions by patients according to RECIST1.1 criteria is shown in the waterfall plot in Figure 4.

**TABLE 3:** Intra-hepatic tumor responses evaluated by RECIST1.1 and mRECIST criteria.

Response	RECIST1.1			mRECIST		
	HAIC group (*n* = 75)	SC group (*n* = 71)	*p*-value	HAIC group (*n* = 75)	SC group (n = 71)	*p*-value
CR	0	0	–	2 (2.7%)	0	-
PR	30 (40%)	12 (16.9%)	–	32 (42.6%)	15 (21.1%)	-
SD	36 (48%)	51 (71.8%)	–	32 (42.6%)	48 (67.6%)	-
PD	9 (12%)	8 (11.2%)	–	9 (26.7%)	8 (31%)	-
ORR	30 (40%)	12 (16.9%)	0.002	34 (45.3%)	15 (21.1%)	0.002
DCR	66 (88%)	63 (88.7%)	0.89	66 (88%)	63 (88.7%)	0.89

**Abbreviations:** HAIC, hepatic arterial infusion chemotherapy; SC, systemic chemotherapy; CR, complete response; PR, partial response; SD, stable disease; PD, progressive disease; ORR, objective response rate; DCR, disease control rate.

### Adverse events and safety

In general, the SC resulted in more AEs than those in HAIC (Table 4). The frequencies of rash (3 [4%] vs. 20 [28.2%]; *p* < 0.001), vomiting (27 [36%] vs. 51 [71.8%]; *p* < 0.001), fatigue (19 [25.3%] vs. 35 [49.3%]; *p* < 0.001), leukopenia (9 [12%] vs. 20 [28.2%]; *p* = 0.014), anemia (13 [17.3%] vs. 33 [46.5%]; *p* < 0.001), and sensory neuropathy (9 [12%] vs. 18 [25.4%]; *p* = 0.038) were lower in the HAIC group. Meanwhile, the overall incidence of serious AEs was higher in the SC group than that in the HAIC group. The frequencies of grades 3–4 vomiting (1 [1.3%] vs. 8 [11.2%]; *p* = 0.032), leukopenia (0 [0] vs. 5 [7%]; *p* = 0.025), and anemia (0 [0] vs. 6 [8.5%]; *p* = 0.012) were significantly higher in the SC group than those in the HAIC group. There were no significant differences in the frequencies of fever (15 [20%] vs. 10 [14.1%]; *p* = 0.343), abdominal pain (19 [25.3%] vs. 13 [18.3%]; *p* = 0.305), diarrhea (2 [2.7%] vs. 2 [2.8%]; *p* = 1.000), neutropenia (6 [8%] vs. 9 [12.7%]; *p* = 0.352), thrombocytopenia (8 [10.7%] vs. 16 [22.5%]; *p* = 0.053), elevated ALT (20 [26.7%] vs. 16 [22.5%]; *p* = 0.563), elevated AST (30 [40%] vs. 24 [33.8%]; *p* = 0.438), hyperbilirubinemia (12 [16%] vs. 10 [14.1%]; *p* = 0.746), hypoalbuminemia (37 [49.3%] vs. 34 [47.9%]; *p* = 0.861), and elevated creatinine (8 [10.7%] vs. 6 [8.5%]; *p* = 0.649). In the HAIC group, three (4%) patients delayed and discontinued treatment because of AEs. In the SC group, seven (9.86%) patients delayed and discontinued the treatment because of AEs.

**TABLE 4:** Objective treatment-related adverse events.

	Any grade			Grades 3–4		
Adverse event	HAIC group (*n* = 75)	SC group (*n* = 71)	*p*-value	HAIC group (*n* = 75)	SC group (*n* = 71)	*p*-value
Rash	3 (4%)	20 (28.2%)	<0.001	0	0	–
Fever	15 (20%)	10 (14.1%)	0.343	0	0	–
Abdominal pain	19 (25.3%)	13 (18.3%)	0.305	3 (4%)	0	0.245
Vomiting	27 (36%)	51 (71.8%)	<0.001	1 (1.3%)	8 (11.2%)	0.032
Fatigue	19 (25.3%)	35 (49.3%)	0.003	0	0	–
Diarrhea	2 (2.7%)	2 (2.8%)	1.000	0	0	–
Leukopenia	9 (12%))	20 (28.2%)	0.014	0	5 (7.0%)	0.025
Neutropenia	6 (8%)	9 (12.7%)	0.352	1 (1.3%)	4 (5.6%)	0.331
Anemia	13 (17.3%)	33 (46.5%)	<0.001	0	6 (8.5%)	0.012
Thrombocytopenia	8 (10.7%)	16 (22.5%)	0.053	0	3 (4.2%)	0.112
Elevated ALT	20 (26.7%)	16 (22.5%)	0.563	1 (1.3%)	1 (1.4%)	1.000
Elevated AST	30 (40%)	24 (33.8%)	0.438	2 (2.7%)	2 (2.8%)	1.000
Hyperbilirubinemia	12 (16%)	10 (14.1%)	0.746	2 (2.7%)	1 (1.4%)	1.000
Hypoalbuminemia	37 (49.3%)	34 (47.9%)	0.861	0	1 (1.4%)	0.486
Elevated creatinine	8 (10.7%)	6 (8.5%)	0.649	0	0	–
Sensory neuropathy	9 (12%)	18 (25.4%)	0.038	0	0	–

Some patients may have multiple immune-related adverse events.

**Abbreviations:** HAIC, hepatic arterial infusion chemotherapy; SC, systemic chemotherapy; ALT, alanine aminotransferase; AST, aspartate aminotransferase.

## Discussion

It is widely acknowledged that iCCA is a gastrointestinal adenocarcinoma with a high level of malignancy and poor prognosis. In addition, most of the patients with iCCA cannot receive surgery because of advanced disease in iCCA, and these patients with unresectable iCCA undergo chemotherapy to control tumor development. Over the past years, GEMCIS and GEMOX have become the standard first-line chemotherapy regimen (Okusaka et al., 2010; Valle et al., 2010; Fiteni et al., 2014; Grenader et al., 2015). However, the occurrence of AEs is an urgent problem to be solved for SC. There is also an urgent need to find a regimen to reduce the occurrence of AEs while achieving similar survival benefits. Localized arterial treatment such as HAIC, TACE, and transarterial radioembolization (TARE) might be important treatment options for advanced cholangiocarcinoma (Mosconi et al., 2021; Ishii et al., 2022; Schaarschmidt et al., 2023). A previous study clarified that patients receiving TARE as first-line therapy had a 68.6% disease control rate and a median OS of 12 months (Schaarschmidt et al., 2023). In addition, a systemic review and meta-analysis demonstrated that the median OS after TACE was 14.2 months, while after TARE, it was 13.5 months for advanced iCCA (Mosconi et al., 2021). Meanwhile, few previous studies indicated that HAIC combined with systemic gemcitabine (GEM) and oxaliplatin may be an effective therapy for patients with advanced iCCA (Marumoto et al., 2014; Cercek et al., 2020). A retrospective study indicated the mFOLFOX regimen used in HAIC could be a new option for patients with iCCA (Cai et al., 2021). Some prospective studies demonstrated that HAIC with mFOLFOX had relatively low toxicity for hepatocellular carcinoma (HCC) (He et al., 2019; Li et al., 2022; Lyu et al., 2022; Li et al., 2023). Although these studies focused on HCC patients, the safety of HAIC with mFOLFOX was still of clinical significance for patients with iCCA, and HAIC with FOLFOX might be a feasible and promising regimen for treating iCCA patients.

In the current study of 146 patients, we compared HAIC with the first-line SC (GEMCIS and GEMOX) and found that patients in the HAIC group had significantly longer IPFS than patients in the SC group and that HAIC showed an ORR higher than SC. In subgroup analyses, single-tumor patients appeared to benefit from considering HAIC in terms of OS and PFS, indicating that HAIC might have a better efficacy than SC in relatively early-stage unresectable iCCA patients and that HAIC could control liver lesions better than SC. One potential explanation for this is that HAIC can provide higher concentrations of the chemotherapeutic agents in the liver than SC, therefore contributing to control tumor in the liver. As is known to all, the liver possesses a dual blood supply. In detail, the hepatic artery provides nearly all of the tumor’s blood flow, and the portal vein supplies blood to the non-neoplastic liver parenchyma. HAIC could preferentially deliver more chemotherapeutic agents to the hepatic artery, which contributes to controlling tumors in the liver.

We also found that patients with unresectable iCCA had similar OS and PFS after HAIC or SC treatment, suggesting that HAIC had a similar clinical efficiency to SC in the outcomes of patients. Although HAIC could better control intrahepatic tumors compared to SC, there were no significant differences in the outcome of patients. It could be explained by the fact that in this study, most patients were at the advanced stage and had extrahepatic metastases. The progression of extrahepatic lesions resulted in the death of patients, and HAIC had a poor control effect on extrahepatic lesions. Therefore, it would be an excellent clinical treatment strategy to add immune therapy and targeted therapy or SC on the basis of HAIC for those patients with extrahepatic metastasis.

Safety and the incidence of AEs are also important indicators for evaluating the chemotherapy regimen apart from the therapeutic effect. The common objective treatment-related AEs observed in this study were rash, fever, abdominal pain, vomiting, fatigue, diarrhea, leukopenia, neutropenia, anemia, thrombocytopenia, elevated ALT, elevated AST, hyperbilirubinemia, hypoalbuminemia, elevated creatinine, and sensory neuropathy. In general, the ratio of AEs in the HAIC group was lower than that in the SC group. The frequencies of rash, vomiting, fatigue, leukopenia, anemia, and sensory neuropathy were also lower in the HAIC group. Hematologic toxicity and liver function damage were the main grade 3-4 AEs in this study. In addition, the frequencies of grade 3–4 AEs were lower in the HAIC group. One possible reason for this is that HAIC enables the delivery of chemotherapy drugs directly into the liver, causing a relatively low systemic blood concentration of drugs. However, SC is the intravenous administration of chemotherapy drugs. In order to achieve the effect of killing liver tumors, the systemic blood concentration of the drug must be at a high level to cause damage to various systems in the body. It is also possible that the liver could clear the drugs via first-pass metabolism to approach diminish systemic toxic effects (Ensminger and Gyves, 1983; Cohen and Kemeny, 2003; Cercek et al., 2020). Meanwhile, most of these AEs were controlled after symptomatic treatment for the HAIC group and would not affect the next session. Therefore, HAIC may be a safe and effective therapeutic regimen for treating patients with unresectable iCCA.

This study also had few limitations. First, it was a retrospective study, and all of the patients came from a single center; thus, further prospective, large-sample, and randomized studies are needed to confirm our findings. Second, the relatively small sample size was limited by the generalizability of our results, and there was a risk of type II error. Finally, more bench-scale research studies are needed to determine the intrinsic mechanism guiding HAIC for patients with iCCA.

In conclusion, this study demonstrated that HAIC was a safe and effective therapeutic regimen in the cohort of 146 patients with unresectable iCCA. Meanwhile, our study indicated that patients with single tumor are most likely to benefit from HAIC than SC.

## Data Availability

The raw data supporting the conclusion of this article will be made available by the authors, without undue reservation.

## Supplementary material

SUPPLEMENTARY FIGURE S1 Median follow-up times of the **(A)** HAIC and **(B)** SC groups calculated by the reversed Kaplan–Meier method.

SUPPLEMENTARY FIGURE S2 Overall survival and progression-free survival of the two groups of patients. Kaplan–Meier curves of **(A)** overall survival and **(B)** progression-free survival for patients in the HAIC, GEMCIS, and GEMOX groups.

SUPPLEMENTARY FIGURE S3 Subgroup analysis for the overall survival and progression-free survival of the two groups of patients. Kaplan–Meier curves of **(A)** overall survival and **(B)** progression-free survival for patients with single tumor.
