# Supplementary figures and images for: Comparison of hepatic arterial infusion chemotherapy with mFOLFOX vs. first-line systemic chemotherapy in patients with unresectable intrahepatic cholangiocarcinoma
Source: Front Pharmacol. 2023 Sep 5;14:1234342. doi: 10.3389/fphar.2023.1234342 (PMC10508288; doi:10.3389/fphar.2023.1234342)

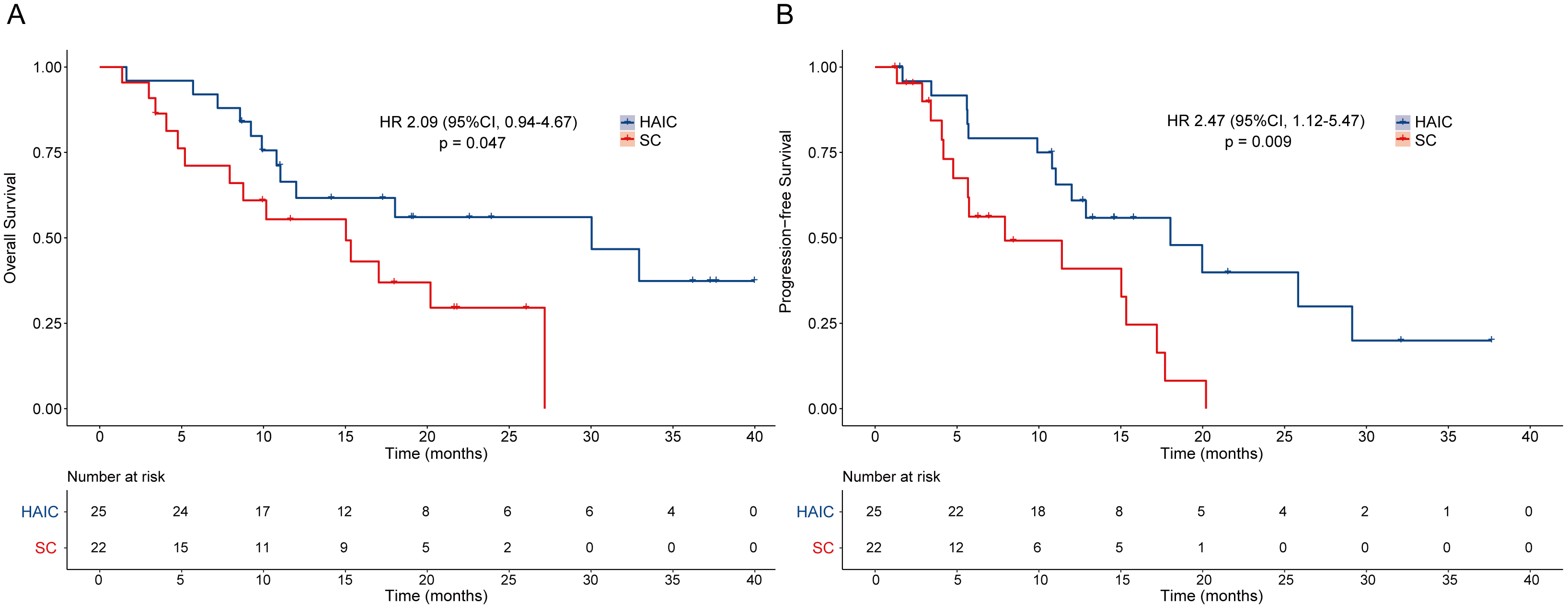

Supplement: Supplementary file 1 [file Image3.TIF]

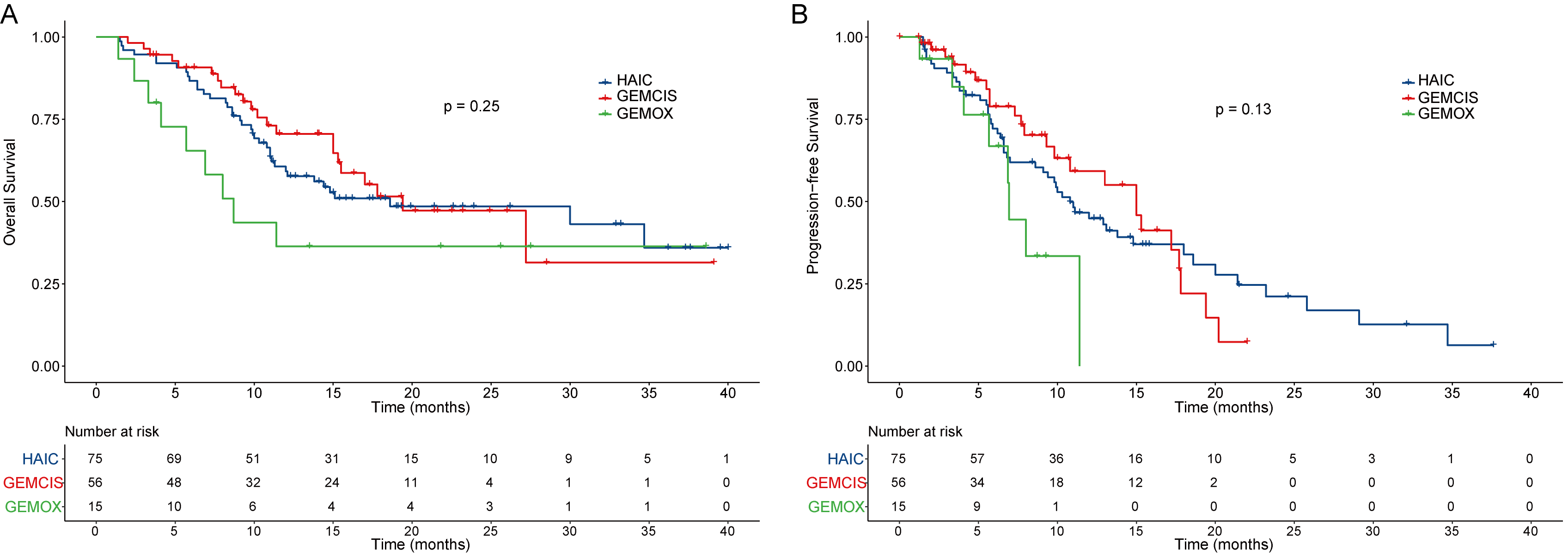

Supplement: Supplementary file 2 [file Image2.TIF]

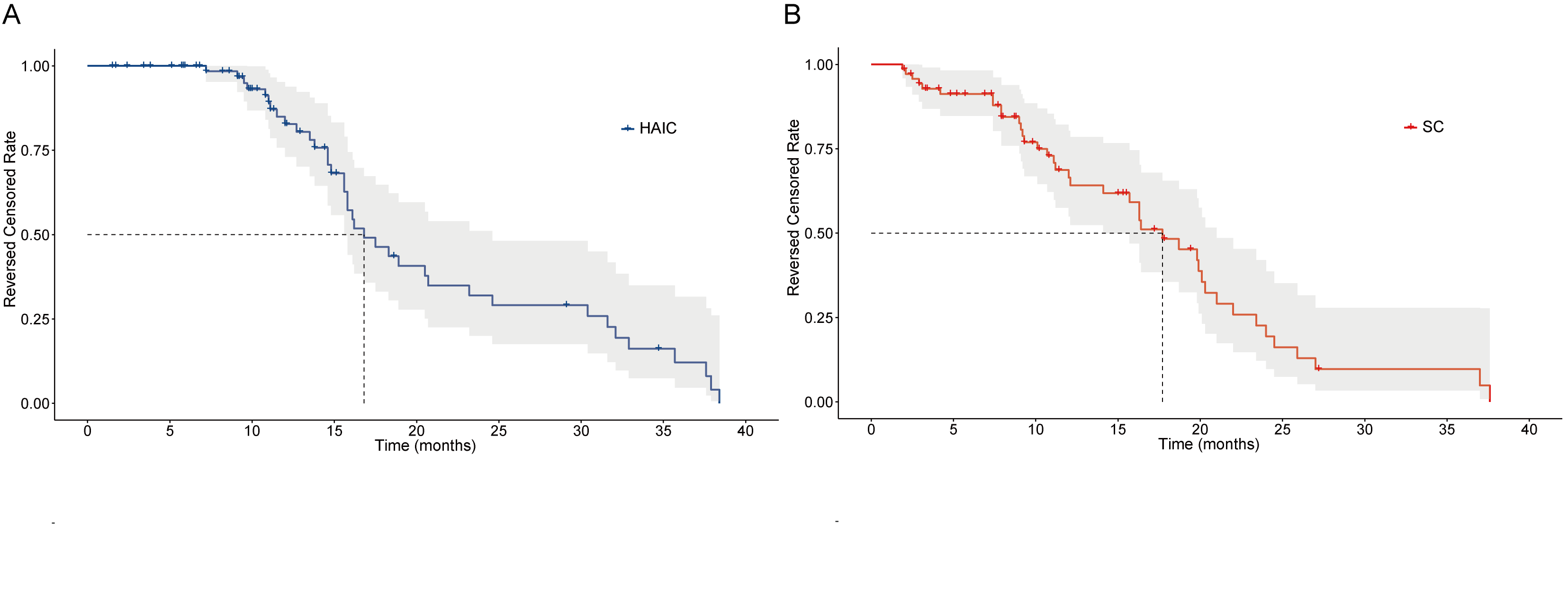

Supplement: Supplementary file 3 [file Image1.TIF]
